# Effects of LPS on the Secretion of Gonadotrophin Hormones and Expression of Genes in the Hypothalamus-Pituitary-Ovary (HPG) Axis in Laying Yangzhou Geese

**DOI:** 10.3390/ani10122259

**Published:** 2020-11-30

**Authors:** Shijia Ying, Jialin Qin, Zichun Dai, Hao An, Huanxi Zhu, Rong Chen, Xiaojin Yang, Wenda Wu, Zhendan Shi

**Affiliations:** 1 Jiangsu Key Laboratory for Food Quality and Safety-State Key Laboratory Cultivation Base of Ministry of Science and Technology, Jiangsu Academy of Agricultural Sciences, Nanjing 210014, China; xuanzaizhu@163.com (H.Z.); chenrong_big@163.com (R.C.); 2 Institute of Animal Science, Jiangsu Academy of Agricultural Sciences, Nanjing 210014, China; d1822863210@163.com; 3 College of Veterinary Medicine, Nanjing Agricultural University, Nanjing 210095, China; hsyqjl@sina.com (J.Q.); 2019807162@njau.edu.cn (H.A.); yangxj@njau.edu.cn (X.Y.); wuwenda@njau.edu.cn (W.W.)

**Keywords:** goose, LPS, gonadotrophin hormone, steroidogenesis, HPG axis

## Abstract

**Simple Summary:**

Lipopolysaccharide (LPS), an endotoxin from *E. coli*, has been proven to impair follicle development and steroidogenesis, secretion of pituitary and hypothalamus reproductive hormones in mammals. However, the effects of LPS on the avian reproductive axis remain elusive. Pathogenic bacterial infection due to the particular mating behavior on the water containing pathogens was reported to decrease the laying rate and cause economic loss in goose production. In this study, we showed that LPS infection disturbed the plasma pituitary gonadotrophin hormone concentrations and the gene expression of the reproductive axis in Yangzhou geese. Notably, for the first time we proved that both the expression of gonadotrophin-releasing hormone (*GnRH)* and gonadotropin-inhibiting hormone (*GnIH*), two important reproductive genes from the hypothalamus, were altered after LPS treatment in birds. Our results can explain the decreased laying rate in goose after bacterial infection, and also provide new insights into reproductive dysfunction caused by LPS and the immune challenge in birds.

**Abstract:**

Lipopolysaccharide (LPS) from gram-negative bacteria was found to be involved in the decrease in laying performance in goose flocks with high stocking density during summer months. LPS injection delayed the increase in the laying rate and altered hierarchical follicle morphology. While there is evidence that LPS exerts suppressive effects on goose reproduction, the time course effects of LPS on the hypothalamus-pituitary-ovary (HPG) axis remain elusive. In this study, we investigated the expression of genes in the HPG axis and the plasma gonadotrophin hormone concentrations in breeding geese at 0, 6, 12, 24, and 36 h after intravenous injection with LPS. The results showed that LPS treatment enhanced and suppressed expression of hypothalamic gonadotropin-inhibiting hormone (*GnIH*) and gonadotrophin-releasing hormone (*GnRH*) mRNA, respectively, and similar effects were observed on the mRNA expression of their receptors, *GnIHR* and *GnRHR*, in the pituitary. LPS treatment transiently increased follicle *FSHβ* mRNA expression at 12 h and exerted no significant effect on *LHβ* mRNA expression in the pituitary. Regardless of the expression of *FSHβ* and *LHβ*, plasma follicle stimulating hormone (FSH) and luteinizing hormone (LH) concentrations were significantly increased during 24–36 h after LPS treatment. In the ovary, *StAR* and *Cyp11a1* were mainly expressed in the granulosa layer (GL) of hierarchical follicles, while *Cyp17a1* and *Cyp19a1* were mainly expressed in white follicles (WFs) and yellowish follicles (YFs), and to a lesser extent in the theca layer (TL). After LPS treatment, the mRNA levels of *Cyp11a1* in the GLs, *Cyp17a1* in the WFs and TL, and *Cyp19a1* in the WFs, YFs, and TL were significantly decreased. However, LPS treatment transiently upregulated *StAR* expression at 12 h. These results indicate that the exposure of laying geese to LPS may impair the HPG axis and disturb ovarian steroidogenesis. Our research provides new insights into reproductive dysfunction caused by LPS and the immune challenge in birds.

## 1. Introduction

In China, the application of the out-of-season laying technique developed by our team balanced year-round goose production and made laying possible in hot summer seasons [1]. The high economic benefits of the out-of-season laying technique has stimulated farmers to enlarge flock sizes or improve stocking densities [2]. We have proven that high stocking density during summer months resulted in higher concentrations of total bacteria, including *Escherichia coli* and *Salmonella* in water, as well as an increase in lipopolysaccharide (LPS) concentrations in water and goose plasma [3]. In geese, the ovary is susceptible to pathogenic bacterial infection due to the particular mating behavior on the water containing pathogens [4]. The higher plasma LPS concentrations and direct ovary infection by bacteria were accompanied by reduced laying performance in geese [3,5].

Avian egg-laying is an orderly and progressive event composed of several processes, including primordial follicle recruitment, pre-hierarchical follicle selection, ovulation of the largest hierarchical follicle (F1), and oviposition [6]. In Magang goose, it takes approximately 18 days for large white follicles (WFs) to develop into the largest hierarchical follicle and another 2 days to be laid as an egg [7]. The reproductive processes in female birds are coordinated by the hypothalamus-pituitary-ovary (HPG) axis. The hypothalamus serves as an integration center that coordinates the activation and inhibition of the axis by releasing neuropeptides in the portal vascular system. Gonadotrophin-releasing hormone (GnRH) and gonadotropin-inhibiting hormone (GnIH) from the hypothalamus stimulate and inhibit the synthesis and release of gonadotropin hormones, i.e., follicle stimulating hormone (FSH) and luteinizing hormone (LH), in the pituitary. FSH and LH in turn support ovarian function, such as follicle development and steroidogenesis. Estradiol (E2) and progesterone (P4) are the two main steroid hormones secreted by follicles, and they regulate follicular development, atresia, and ovulation [6]. E2 and P4 also play important roles in negative and positive feedback regulation of the HPG axis [6]. In birds, the LH surge before ovulation was found to be positively regulated by P4 rather than E2 in mammals [6]. Disruption of the HPG axis by infection with an environmental pathogen and immune stress would impair avian reproduction processes and result in decreased laying performance.

LPS is derived from gram-negative bacteria, and it has been reported to impair reproductive performance in both mammals and birds [8,9]. Toll-like receptors (TLRs), the receptors of LPS, are expressed in follicles of various sizes, which means that LPS may directly inhibit follicle development and steroidogenesis [8,9]. In mammals, such as mice and bovines, exposure to LPS in vivo or in vitro resulted in a diminished follicular reserve in the ovary [10], and the exposure impaired E2 and P4 synthesis from cultured follicular granulosa cells [11]. In bovines, follicles with high levels of LPS (> 0.5 EU/mL) had lower E2 than follicles with lower LPS concentrations [12]. In addition, evidence also indicates that LPS suppresses ovarian function by decreasing pulsatile GnRH secretion and decreasing pituitary hormone synthesis and secretion in mammals [13]. In gonadectomized animals, an LPS challenge suppressed GnRH release and disrupted the LH surge amplitude, frequency, and concentration [14,15,16,17,18]. In agreement with reduced E2 compromising ovulation, when LPS was infused into the uterine lumen, the preovulatory LH surge was attenuated [19]. Furthermore, LPS-treated females had delays in the time to LH surge [20] and lower ovulation rates [21]. However, unlike studies in mammals, most LPS-challenging studies in birds have focused on host immune responses [22,23,24,25], and the effects of LPS on the HPG axis of birds remain largely elusive. In our previous study with Yangzhou geese and Magang geese, we proved that LPS injection significantly decreased the plasma E2 and P4 concentrations [4], which may partially explain the decrease in laying performance after LPS challenge. Until now, the only study in birds that assessed the acute effects of LPS injection (3 h) on GnRH and GnIH expression was performed with zebra finches [26], and no time-course effects of LPS on hypothalamic and pituitary hormone synthesis and secretion in birds have been reported.

Therefore, this study was designed to determine the time course effects of LPS on the expression of genes in the HPG axis and gonadotrophin secretion in geese and to unravel the mechanisms of how pathogen exposure or LPS challenge suppresses goose laying performance from an endocrinology perspective.

## 2. Materials and Methods

### 2.1. Animals and Experimental Design

The animals and experimental design were the same as previously reported [4]. In brief, Yangzhou geese of approximately 540 days of age at peak laying were kept under natural photoperiod and were provided with feed and water ad libitum. The laying behavior of the birds was monitored using a computer video system [7]. The average oviposition interval of the geese was 46.8 h [7]; geese in this experiment were injected with LPS from *E. coli* 055:B5 (Sigma, Shanghai, China) (1.5 mg/kg BW) at −28 h (*n* = 5), −16 h (*n* = 5), −4 h (*n* = 5), 2 h (*n* = 5), and 8 h (*n* = 10) relative to oviposition, and then they were slaughtered 8 h after oviposition by cervical dislocation. This means that the sampling times were 36, 24, 12, 6, and 0 h after LPS injection. All experimental procedures were approved by the Institutional Animal Care and Use Committee of the Jiangsu Academy of Agricultural Sciences (SYXK(Su)2015-0020).

### 2.2. Blood Collection

Blood samples (2 mL) were collected from each bird by wing vein puncture and then were placed in ice-cold heparinized tubes immediately before slaughter. Serum was separated by centrifugation at 4 °C, and then it was stored at −20 °C until assay.

### 2.3. Hormone Assays

Plasma FSH and LH concentrations were measured by sandwich enzyme-linked immunosorbent assay (ELISA) kits according to the manufacturer’s protocols. The sensitivity of the FSH ELISA kit (JL21761, Jianglai Bio, Shanghai, China) and LH ELISA kit (JL21772, Jianglai Bio, Shanghai, China) was 0.1 mIU/mL and 1.0 ng/mL, respectively. Inter- and intra-assay coefficients of variation for both FSH and LH assays were less than 9% and 11%, respectively. Each sample was measured in triplicate.

### 2.4. Tissue Collection

Hypothalamus tissue samples, pituitary tissue samples, white follicles (WFs), yellowish follicles (YFs), theca layer (TL), and granulosa layer (GL) of the first largest to the fifth largest hierarchical follicles (F1–F5) were isolated and snap-frozen in liquid nitrogen. Notably, the theca and granulosa layers of hierarchical follicles that became an irregular ellipse or circle in shape and became deep yellow in appearance after LPS treatment for 24 or 36 h were not isolated, since the yolks were gelatinous and could not be drained out, which resulted in failure to separate the granulosa and theca layers of the follicles. Thus, these denatured hierarchical follicles (DFs) were directly snap-frozen in liquid nitrogen.

### 2.5. RNA Isolation and RT-qPCR

Total RNA was extracted using an RNAprep Pure Tissue Kit (Tiangen, Beijing, China), and it was reverse-transcribed to generate cDNA using PrimeScript RT Master Mix (TaKaRa, Dalian, China) according to the manufacturer’s instructions. RT-qPCR was performed on an ABI 7500 system (Applied Biosystems, Shanghai, China) using FastStart Universal SYBR Green Master Mix (ROX; Roche Diagnostics). The primers used in RT-qPCR are listed in Table 1. Gene expression levels were calculated using the 2^−∆∆CT^ method and were normalized to *β-actin* mRNA expression.

### 2.6. Statistical Analysis

Statistical analysis was performed using the SPSS statistical software program (Version 13.0; SPSS). One-way ANOVA along with Duncan’s multiple tests were applied to analyze the differences in the gene expression of the HPG axis and plasma LH and FSH concentrations after LPS treatment. The results are expressed as the mean ± s.e.m., and the differences were considered significant at *p* < 0.05.

## 3. Results

### 3.1. Effects of LPS on Plasma Gonadotrophin Hormone Levels

**Figure 1:**
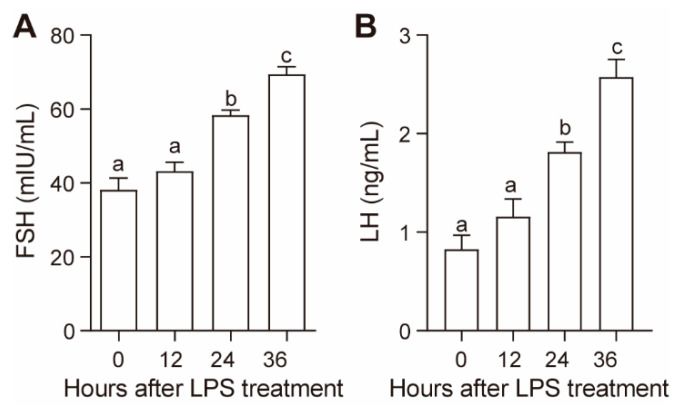
Effect of lipopolysaccharide (LPS) on plasma follicle stimulating hormone (FSH) (**A**) and luteinizing hormone (LH) (**B**) concentrations. Values with different letters are significantly different (*p* < 0.05).

LPS treatment increased plasma FSH and LH concentrations in a time-dependent manner (Figure 1). Both the plasma FSH and LH concentrations significantly increased at 24 h (*p* < 0.05) and further increased at 36 h after LPS administration (*p* < 0.05) (Figure 1).

### 3.2. Effect of LPS on mRNA Expression of Hypothalamic Reproductive Genes

**Figure 2:**
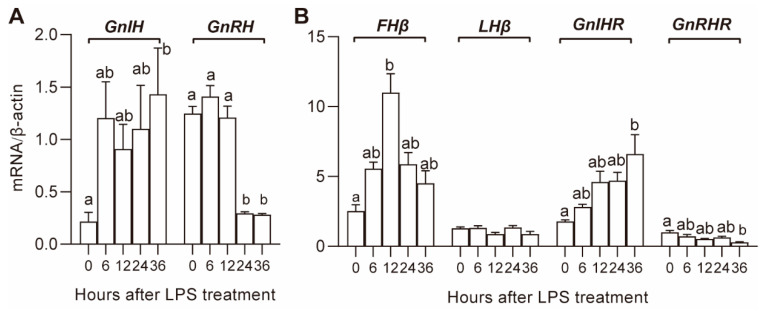
Effect of LPS on gene expression in the hypothalamus (**A**) and pituitary (**B**). Values with different letters are significantly different (*p* < 0.05).

The expression of the hypothalamic reproductive genes *GnRH* and *GnIH* after LPS treatment is shown in Figure 2A. The mRNA expression of *GnRH* significantly decreased at 24 and 36 h after LPS treatment; the expression of *GnIH* tended to increase at 6 h after LPS treatment, and the increase was significant at 36 h.

### 3.3. Effect of LPS on mRNA Expression of Pituitary Reproductive Genes

The expression of pituitary gonadotrophin hormone genes, *FSHβ*, *LHβ,* and receptors of hypothalamic hormones, *GnRHR* and *GnIHR,* is shown in Figure 2B. The expression of *GnRHR* and *GnIHR* tended to decrease and increase, respectively, and the decrease and increase became significant (*p* < 0.05) at 36 h. The expression of *FSHβ* increased in the first 12 h and then gradually decreased until the end of the experiment. There was no significant effect of LPS on the expression of *LHβ*.

### 3.4. Effect of LPS on Ovarian Reproductive Gene Expression

**Figure 3:**
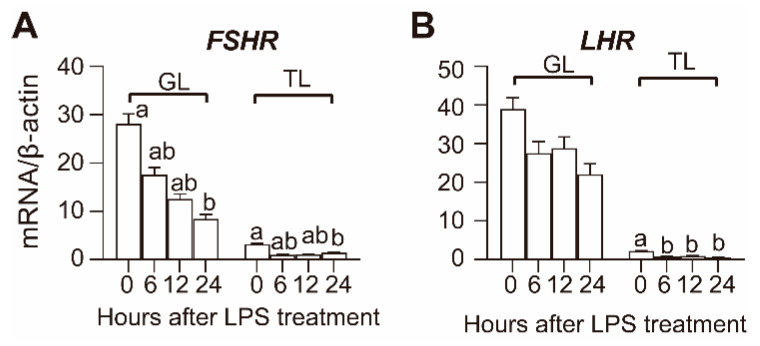
Effect of LPS on FSHR (**A**) and LHR (**B**) mRNA expression in hierarchical follicles. GL, granulosa layer; TL, theca layer. Values with different letters are significantly different (*p* < 0.05).

The transcript levels of gonadotropin hormone receptors in the GL and TL of the hierarchical follicles after LPS treatment are shown in Figure 3. In hierarchical follicles, both the transcript abundance of *FSHR* and *LHR* were relatively higher in GL than they were in TL, and LPS treatment decreased the mRNA levels of *FSHR* and *LHR* in both GL and TL. The *FSHR* transcript was significantly decreased in GL during the 24–36 h period after LPS treatment, while the decrement in TL was only significant at 36 h. The *LHR* transcript was significantly decreased in GL at 36 h after LPS treatment, and the decrement in TL was significant beginning at 6 h after LPS treatment.

### 3.5. Effect of LPS on the Gene Expression of Steroidogenesis Proteins in Follicles

**Figure 4:**
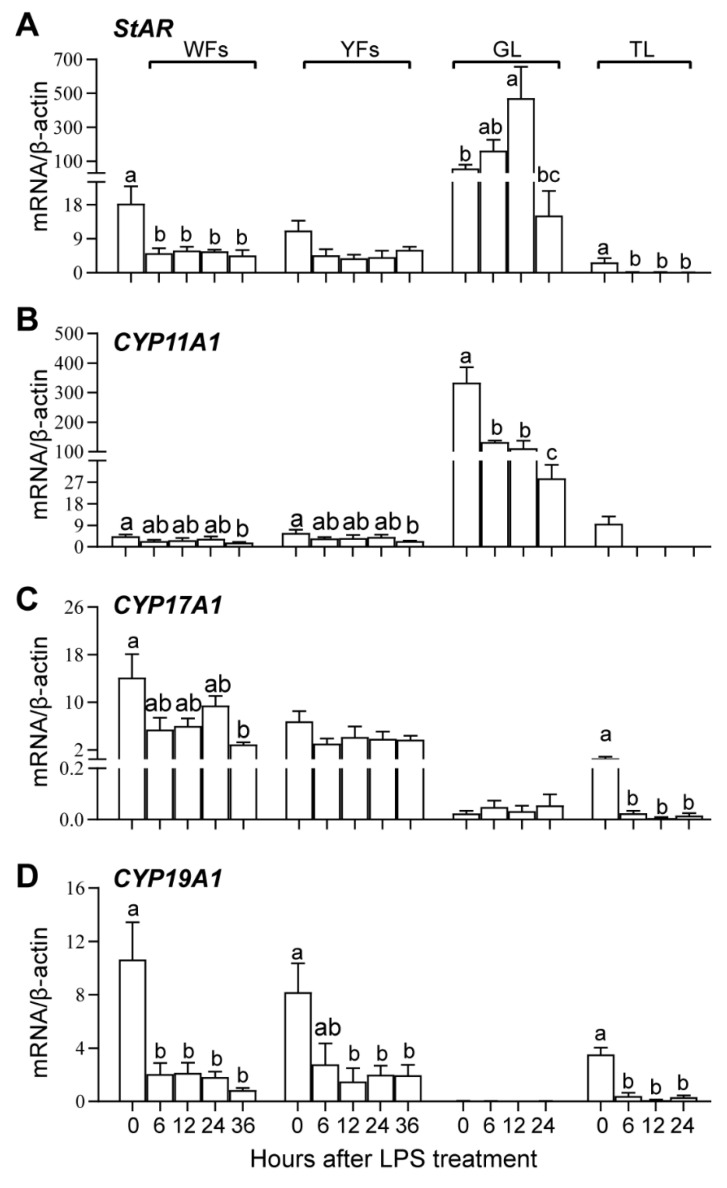
The gene expression of steroidogenic proteins StAR (**A**), Cyp11a1 (**B**), Cyp17a1 (**C**) and Cyp19a1 (**D**) during follicular growth and after LPS treatment. WFs, white follicles; YFs, yellowish follicles; GL, granulosa layer of the hierarchical follicles; TL, theca layer of the hierarchical follicles. Values with different letters are significantly different (*p* < 0.05).

The transcript levels of ovarian steroidogenesis proteins in WFs, YFs, and hierarchical follicles after LPS treatment are shown in Figure 4. The TL and GL were not isolated from DFs; hence, the time-course effects of LPS treatment on the mRNA expression of steroidogenesis proteins were not analyzed in the TL and GL at 36 h after LPS stimulation.

In the control condition (0 h), *StAR* and *Cyp11a1* are mainly expressed in the GL of hierarchical follicles, and their expression is relatively lower in the WFs, YFs, and TLs of hierarchical follicles. *Cyp17a1* and *Cyp19a1* are mainly expressed in the WFs, YFs, and TL.

In WFs, LPS treatment significantly decreased the mRNA expression of *StAR* and *Cyp19a1* at 6 h and only significantly decreased the expression of *Cyp11a1* and *Cyp17a1* at 36 h. In YFs, LPS treatment significantly decreased the transcript abundance of *Cyp11a1* and *Cyp19a1* at 36 h and 12–36 h, respectively. In GL, the transcript abundance of *StAR* was significantly increased at 12 h, while the expression of *Cyp11a1* was decreased after 6 h of LPS treatment. In TL, the expression of *StAR*, *Cyp17a1,* and *Cyp19a1* was decreased since 6 h after LPS treatment. LPS treatment showed no significant effects on *StAR* and *Cyp17a1* expression in YFs, *Cyp17a1* expression in GL, and *Cyp11a1* in TL. These results suggest that LPS treatment inhibited steroid synthesis by suppressing the expression of *Cyp11a1*, *Cyp17a1,* and *Cyp19a1* rather than *StAR* in GL.

## 4. Discussion

In this study, we proved that LPS administration altered the HPG axis and suppressed ovarian steroidogenesis in laying geese. We demonstrated that LPS administration stimulated *GnIH* mRNA expression, indicating that GnIH neurons, such as previously implicated GnRH neurons, were important targets of LPS in the hypothalamus in birds. Notably, the plasma FSH and LH concentrations were increased after LPS injection, and LPS suppressed ovarian steroidogenesis by decreasing the expression of gonadotropin receptors and steroidogenic proteins. To the best of our knowledge, this is the first study focusing on the endocrine mechanism underlying the suppressive effect of LPS on avian reproduction performance.

The current study clearly showed that *StAR* and *Cyp11a1* were mainly expressed in the GL of hierarchical follicles, while *Cyp17a1* and *Cyp19a1* were mainly expressed in WFs and YFs and to a lesser extent in the TL of hierarchical follicles. This is consistent with the knowledge that P4 is mainly produced by the GL of hierarchical follicles, and E2 and testosterone (T) are mainly produced by nonhierarchical follicles and the TL of hierarchical follicles [27,28,29,30]. The GL became steroidogenically active and started to express *StAR* and *Cyp11a1* during the transition of the YFs to preovulatory hierarchy follicles, which is stimulated initially by FSH and then by LH [6,31,32]. P4 that is predominantly synthesized in the GL is transported to the TL, where it is initially converted to testosterone (T), which is eventually metabolized to E2 by Cyp19a1 [33].

LPS was reported to suppress ovarian steroidogenesis and the expression of steroidogenic proteins [4,8,34,35]. Woods et al. [34] reported that treatment with LPS attenuated agonist-induced progesterone synthesis in undifferentiated chicken granulosa cells in vitro. In our previous publication, we demonstrated that LPS injection resulted in a time-dependent decrease in the plasma P4 and E2 concentrations in geese [4]. The decrease in P4 secretion may be explained by the reduction in the gene expression of *Cyp11a1* in GL, while the decrease in E2 secretion may be explained by the reduction in *Cyp17a1* and *Cyp19a1* mRNA abundance in follicles. Unexpectedly, the expression of *StAR*, a rate-limiting protein in P4 synthesis that mediates the transport of cholesterol from outside of the mitochondrial membrane to the inside mitochondrial membrane, was upregulated in the GL at 24 h after LPS injection. This result is reminiscent of a report that LPS treatment upregulated *StAR* mRNA expression in duck Leydig cells in vitro [36]. Although goose granulosa cells and duck Leydig cells are two different cell types, LPS may exert the same effects on *StAR* gene expression in these two avian gonadal cell types. These effects of LPS on *StAR* mRNA expression may also be explained by the follicle development stage. Unlike the expression of *StAR* in GL, LPS treatment reduced *StAR* expression in WFs and the TL of hierarchical follicles in the current study. In bovines, higher follicle fluid LPS concentrations tend to increase *StAR* mRNA expression in the TL of follicles >8 mm but not in follicles <8 mm [12]. Regardless, these results suggested that the decrease in plasma P4 concentration may be mainly caused by the decrease in the mRNA expression of *Cyp11a1* rather than *StAR* in the GL.

LPS was reported to suppress cell function by increasing immune stress and oxidative stress through binding to TLR2 and TLR4 [11,37,38,39]. In cultured bovine granulosa cells, LPS suppressed steroidogenesis via the TLR2 and TLR4 pathways [11]. We have recently proven that TLR2 and TLR4 are dynamically expressed during follicle development in laying geese [16]. Thus, LPS may directly suppress goose follicle steroidogenesis in a manner that is similar to what occurs in the granulosa cells of mammals. In addition, the steroidogenesis and expression of steroidogenic proteins were stimulated by LH and FSH through their receptors in the follicles. In the current study, plasma FSH and LH concentrations were upregulated after LPS treatment. However, the mRNA expression of *FSHR* and *LHR* in both the GLs and TLs of hierarchical follicles was significantly decreased, and LPS may suppress the stimulatory effects of gonadotrophin hormones on ovarian steroidogenesis by decreasing the expression of gonadotropin receptors. In bovines, the *FSHR* and *LHR* mRNA abundance in F1 and F2 higher follicle fluids LPS concentrations was significantly reduced and was accompanied by altered E2 and P4 in follicle fluids [40]. The results indicated that LPS may exert the same effects on *FSHR* and *LHR* gene expression in mammals and birds. Unfortunately, *FSHR* and *LHR* mRNA abundance was not detected in nonhierarchical follicles in this study, and whether LPS treatment abolishes the stimulation effects of gonadotrophin remains to be elucidated. Since the expression of *FSHR* and *LHR* are also key markers of follicle selection [6], learning about these effects in the future will be of particular interest. Collectively, LPS suppresses ovarian steroidogenesis in geese by abolishing the stimulation effects of gonadotrophin through decreasing *FSHR* and *LHR* expression or through directly suppressing the expression of steroidogenic proteins.

The hypothalamus serves as an integration center and coordinates the activation and inhibition of the HPG axis by releasing GnRH and GnIH. LPS administration decreased GnRH synthesis [17,18,41] and impaired GnRH-induced LH secretion [17,42,43]. In this study, LPS treatment indeed reduced the expression of *GnRH* in the hypothalamus and *GnRHR* in the pituitary. Unexpectedly, LPS administration gradually increased plasma LH and FSH concentrations. This result is consistent with a report that LPS administration dramatically increased serum LH (after 2 h) and FSH (after 4 h) concentrations in rats [44]. However, an LPS challenge was reported to decrease the secretion of LH in bovines [16], sheep [17], and rats [45,46]. The mechanism underlying the stimulatory effects of LPS treatment on LH and FSH secretion in the current study is not clear. However, the findings are reminiscent of a phenomenon observed in our previous study with out-of-season Magang geese. At the end of the out-of-season laying period, the circulating LH rose until the incubation was established, regardless of the lack of follicle development at this stage [1]. The nonparallel plasma LH concentration and follicle development was thought to be caused by the decline in plasma E2 and P4 concentrations, which may exert negative feedback regulation on gonadotrophin hormone synthesis and release. As ovarian steroidogenesis was significantly influenced, the lack of negative feedback regulation by E2 and P4 may also partially explain the upregulation of LH and FSH release in the current study.

GnIH, also called RFamide-related peptide 3 (RFRP3) in mammals, is involved in stress-induced reproductive dysfunction [13,47,48]. In the current study, an LPS challenge significantly upregulated GnIH and GnIHR expression, indicating that GnIH neurons are important targets of LPS. Consistent with our results, a high dose (rather than a moderate dose) of LPS upregulated GnIH expression in both ovariectomized and gonadal intact female rats [41]. Furthermore, the upregulation of GnIH mRNA levels was negatively correlated with the reduction in GnRH mRNA, indicating that GnIH may play important roles in immune stress-induced reproductive dysfunction. However, there are also reports indicating that GnIH is not involved in LPS-mediated suppression of GnRH expression. Lee et al. [46] proved that the LPS-mediated suppression of GnRH expression is not caused by a change in RFRP1 expression in rats. Similarly, in a study with zebra finches, Lopes et al. [26] reported that LPS injection exerted rapid inhibitory effects on the HPG axis by reducing the GnRH mRNA level, without altering the GnIH mRNA level. These results suggest that GnIH may only participate in the suppression of GnRH under certain conditions. In the future, more analyses are needed to elucidate the roles of GnIH in the LPS-mediated suppression of the HPG axis, and to unravel the underlying mechanisms of reproductive dysfunction caused by LPS.

## 5. Conclusions

We studied the effects of LPS on the HPG axis in laying geese and demonstrated that both GnRH and GnIH are involved in LPS-induced reproductive disorders. An LPS challenge suppressed ovarian steroidogenesis and inhibited the stimulatory effects of gonadotrophin by decreasing the expression of *FSHR* and *LHR* rather than decreasing the plasma FSH and LH concentrations. Our findings provide new insights into how immune challenges influence reproductive performance in geese.

## Figures and Tables

**Table 1:** Primers used in the real-time quantitative PCR of genes in goose samples.

Gene	Primer Sequences (5′–3′)	Accession Number	Length (bp)
β-actin	F: TGACGCAGATCATGTTTGAGA R: GCAGAGCGTAGCCCTCATAG	M26111.1	159
GnRH	F: CTGGGACCCTTGCTGTTTTG R: AGGGGACTTCCAACCATCAC	MT007957.1	132
GnIH	F: ATCTACCTAGGCATGCTCCAA R: ACAGGCAGTGACTTCCCAAAT	KC514473.1	155
VIP	F: ACCAGTGTCTACAGCCATCTTTTG R: AGGTGGCTCAGCAGTTCATCTACA	DQ023159	204
TRH	F: GCAAGAGGGGCTGGAATGAT R: ATGGCAGACTGCTGAAGGTC	NM 001030383.2	133
GnRHR	F: TCTGCTGGACCCCCTACTAC R: TCCAGGCAGGCATTGAAGAG	KJ659046.1	127
GnIHR	F: GTCGTCATGTACACCCGCAT R: TCTTGCGAGACACCTTCCTC	KC514473.1	103
VIPR	F: TACTGCGTCATGGCCAACTT R: TGTCCAAGCGGTGATGAACA	NM 001097523.1	153
TSHR	F: CTATGGCTATGTGGGGTGCC R: ACTGAGGCGAAAGACCAGAC	NM 204930.1	189
FSHβ	F: GTGGTGCTCAGGATACTGCTTCA R: GTGCAGTTCAGTGCTATCAGTGTCA	KC777370.1	209
LHβ	F: GACCCGGGAACCGGTGTA R: AGCAGCCACCGCTCGTAG	DQ023159	90
PRL	F: TGCTCAGGGTCGGGGTTTCA R: GCTTGGAGTCCTCATCGGCAAGTT	DQ023160	218
TSHβ	F: CTCTGTCCCAAAACGTGTGC R: CCACACTTGCAGCTTATGGC	FJ797681.1	121
FSHR	F: AGTTTCCTGCCAGGTCACGG R: CAAGGTCTTGCTTAGCCTGAGA	KC477215.1	214
LHR	F: TCGCTGTGGTCAGCAGAAAA R: AGCTGTACCCCAGGATGTCT	XM_013192443.1	199
ESR1	F: ATGGCAACAACCTTCTGGGAT R: GGTGTGAAGGGTCATGGTCA	XM_013178332.1	117
ESR2	F: GGCAAACGTCAAGCCCAAAT R: CTGGTCACAGGTAGCACTGG	XM_013182959.1	151
Cyp11a1	F: GGCTCAACCTCAACCACTT R: GGGCTTGTTGCGGTAGTC	KY463321.1	60
StAR	F: GGAGCAGATGGGAGACTGGA R: CGCCTTCTCGTGGGTGAT	KF958133.1	60
Cyp17a1	F: CTCACTGACACCAGCATCGG R: GGGCTTGTCCCACTCCTT	XM_013174485.1	102
Cyp19a1	F: TGATTGCTGCTCCTGATA R: GAGAATAATGTTTGTTCCCT	KY763000.1	278
